# Tau Clearance Mechanisms and Their Possible Role in the Pathogenesis of Alzheimer Disease

**DOI:** 10.3389/fneur.2013.00122

**Published:** 2013-09-03

**Authors:** Adrianne S. Chesser, Susanne M. Pritchard, Gail V. W. Johnson

**Affiliations:** ^1^Neuroscience Graduate Program, Department of Anesthesiology, University of Rochester, Rochester, NY, USA

**Keywords:** tau, proteasome, autophagy, proteolysis, degradation

## Abstract

One of the defining pathological features of Alzheimer disease (AD) is the intraneuronal accumulation of tau. The tau that forms these accumulations is altered both posttranslationally and conformationally, and there is now significant evidence that soluble forms of these modified tau species are the toxic entities rather than the insoluble neurofibrillary tangles. However there is still noteworthy debate concerning which specific pathological forms of tau are the contributors to neuronal dysfunction and death in AD. Given that increases in aberrant forms of tau play a role in the neurodegeneration process in AD, there is growing interest in understanding the degradative pathways that remove tau from the cell, and the selectivity of these different pathways for various forms of tau. Indeed, one can speculate that deficits in a pathway that selectively removes certain pathological forms of tau could play a pivotal role in AD. In this review we will discuss the different proteolytic and degradative machineries that may be involved in removing tau from the cell. How deficits in these different degradative pathways may contribute to abnormal accumulation of tau in AD will also be considered. In addition, the issue of the selective targeting of specific tau species to a given degradative pathway for clearance from the cell will be addressed.

## Introduction

Insoluble, fibrillar intraneuronal accumulations of pathological forms of the tau protein called neurofibrillary tangles (NFTs) are important and defining hallmarks of the Alzheimer disease (AD) brain. Indeed, the progression of AD can be neuropathologically staged based on the location and extent of tau pathology (1). The predominant post-translational modification of tau in the NFTs is phosphorylation; however numerous modifications have been noted including truncation, acetylation, nitration, and several others (2–4). Historically the NFTs were considered to be the toxic entities, however over the past decade a new conceptual framework has developed in which pathologically modified monomeric and/or soluble oligomeric forms of tau are considered to be the harmful species (5, 6). Nevertheless, determining exactly which forms of tau compromise neuronal function is still an area of significant investigation. Even though the modifications of tau that are the primary contributors to toxicity have not been conclusively determined, it is clear that tau plays an essential role in the pathogenesis of AD. Given that in animal models of AD reducing tau levels attenuates neuronal dysfunction (7, 8), and in humans the extent of tau pathology correlates with cognitive decline (9), there is a growing interest in defining the degradative pathways that remove tau from the cell. Also of importance is understanding the role of non-degradative cleavage in influencing the eventual clearance of tau. Numerous proteases have been shown to proteolyze tau including aminopeptidases (10–12), thrombin (13–15), human high temperature requirement serine protease A1 (HTRA1) (16), calpain (17–20), and caspases (21–24). Overall, however, most of these enzymes do not appear to be principally responsible for tau clearance. Instead, they are able to generate modified tau species which may then contribute to developing tau pathology, enhanced tau clearance, or both. The bulk of clearance of both physiological and pathological forms of tau is instead mediated by the proteasomal and autophagic degradative systems (25). The contribution of each of these pathways in the turnover of tau, and which forms of tau – including various proteolytic forms – are degraded by each pathway, is an area of significant interest. Our understanding of this issue to date will be reviewed below, and the role of tau proteolysis on subsequent degradation will be discussed. Delineating how these pathways may be compromised in AD and how this contributes to tau pathology is of great importance and could have significance for informing new therapeutic approaches.

## Tau Proteolysis

Tau is a cytosolic, dynamically regulated protein. In differentiated PC12 cells, a pulse-chase experiment showed that ∼90% of the tau was degraded in 18 h (26). Normal, monomeric tau is likely a proteasomal substrate. However, there is evidence that tau is also a substrate for a wide range of proteases as indicated above. This is significant as tau proteolysis could be beneficial in disease by helping to enhance removal of abnormal tau from the cell. Alternately, it could be detrimental by generating toxic fragments. Below we will discuss the different proteases that have been shown to act on tau, at least *in vitro*, and the possible involvement of these proteolytic events in AD.

### Aminopeptidases

Aminopeptidases are a group of enzymes that cleave from the N-terminal end of a protein. The family includes alanyl, arginyl, and glutamyl peptidases. Puromycin sensitive aminopeptidase (PSA) is an alanyl peptidase that is responsible for ∼90% of the aminopeptidase activity in the brain (10). PSA was identified as a potential player in tau pathology through a microarray analysis of gene expression in disease-vulnerable vs. disease-resistant brain regions in JNPL3 mice that overexpress a mutant form of tau (P301L) found in the disease frontotemporal dementia and parkinsonism linked to chromosome 17 (FTDP-17). These mice develop neurodegeneration in the cortex while the cerebellum is relatively spared [although in the original description of these animals pathology was found in the deep cerebellar nuclei (27)]. Interestingly, PSA was found to be elevated in the cerebellum of these TAU^P301L^ mice (10). The levels of PSA are also higher in human cerebellum compared to cortex in both controls and FTD cases. A slight elevation in PSA was also observed in FTD cortices compared to controls. In addition, a non-functional PSA mutant exacerbated tau pathology in a *Drosophila* model of tauopathy, while overexpressing PSA ameliorated the tau phenotype and diminished tau levels (10). Overexpressing PSA had a similar effect in the TAU^P301L^ mice, reducing the pathologic phenotype (delaying paralysis, increasing motor neuron density in the spinal cord, decreasing gliosis) and decreasing tau levels (12). PSA was able to cleave recombinant tau *in vitro*, as well as tau from control human brain (11). However, the data presented in this study suggest that PSA is cleaving tau from both the C- and N-terminal ends, which is not expected from an aminopeptidase. Additionally, other studies failed to demonstrate tau cleavage by PSA (28, 29). One explanation for these discrepancies may be the limitations of *in vitro* assays and experimental techniques. For example, the FTDP-17 mutant tau used in many studies, while relevant for human tauopathy, is not found in AD. Additionally, this form of tau may be processed differently than tau without this mutation. For example, it has been shown that the isomerase Pin1, which has been implicated in AD (30), had opposite effects on P301L and wild-type tau degradation (31). An alternative explanation for the effects of PSA may be that PSA is indirectly regulating tau degradation. PSA has been shown to be involved in the induction of autophagy and specifically the formation of autophagosomes, in a model of overexpressed mutant huntingtin (32). Thus, the *in vivo* effects of PSA on promoting tau clearance may relate to its ability to modulate the key clearance pathway for abnormal and aggregated proteins (to be described in more detail below).

### Thrombin

Thrombin is a serine protease that is a well characterized component of the coagulation cascade. It is typically produced and secreted by endothelial cells, including those in the brain in response to hemodynamic injury. Thrombin may be inappropriately expressed in AD brain. A recent study showed that thrombin is elevated in microvessels isolated from AD brain compared to microvessels from control brain (33). Additionally, thrombin was present in the CSF of AD patients but not in that of controls (33). This is important, as thrombin can act as a neurotoxin by activating intracellular signaling cascades causing neurite retraction and stimulating apoptosis (34–36). Thrombin may also be influencing tau pathology, as treatment of immortalized hippocampal neuronal cells (HT22 cells) with thrombin resulted in the formation of thioflavin-S positive tau aggregates within 24 h, followed by an increase in cell death at 72 h (37). It is unclear how this exogenously applied thrombin may be altering tau within the cells. There are also data to suggest that thrombin may act intracellularly to mediate tau pathology. Thrombin is expressed within neurons and astrocytes in both normal and AD brain (38). In AD brain the staining pattern for thrombin and prothrombin was characteristic of the pattern of NFTs, although these structures were not colabeled with antibodies for tau (38). Evidence supporting a role for thrombin in tau proteolysis came initially from an *in vitro* study showing that thrombin degraded recombinant full-length tau from the N-terminus yielding a 25-kDa fragment, while preserving the microtubule binding repeat domain (13). A later study, however, showed that in N2a neuroblastoma cells expressing a construct of only the tau repeat domain, thrombin cleavage could still occur, indicating additional cleavage sites (15). Similar results were observed in an *in vitro* assay (15).

The products of thrombin proteolysis are potentially pathogenic. Thrombin cleavage of the repeat domain construct yielded fragments that rapidly aggregated, which closely correlated with toxicity in cell culture (15). These fragments can also induce the aggregation of full-length tau (39). A final point of interest relates to potential upstream modifications of tau. Endogenous tau is phosphorylated, and in AD, tau phosphorylation becomes dysregulated. This may interfere with subsequent processes including cleavage and degradation. For example, tau that is in the *cis*-conformation at T231 appears resistant to degradation, as *cis*-tau is found in dystrophic neurites while *trans*-tau is not. Additionally *cis*-tau partitions to the insoluble fraction (30). Phosphorylation at T231 prevents the isomerase Pin1 from converting *cis*-tau to *trans*-tau (30). Interestingly, phosphorylation of tau also appears to disrupt some thrombin cleavage sites, changing the pattern of cleavage without impeding the thrombin-mediated proteolysis (14, 28). It has yet to be determined whether there is a difference in toxicity potential between fragments generated from phosphorylated vs. unphosphorylated tau. Nonetheless, thrombin is a potential candidate for contributing to tau proteolysis and pathology.

### Human high temperature requirement serine protease A1

Another serine protease recently implicated in tau processing is HTRA1. This is a ubiquitously expressed, ATP-independent intracellular protease. Expression is detectable in many tissues, including the nervous system, although expression is low (40). Nonetheless, this enzyme was initially implicated in AD because it may play a role in amyloid processing (41). Tubulin was later identified as a substrate for HTRA1, suggesting HTRA1 may be involved in mediating microtubule function (42, 43). A more recent study showed that HTRA1 can cleave recombinant tau *in vitro* into multiple fragments of varying sizes, and furthermore can degrade insoluble and fibrillarized tau (16). This ability to degrade aggregates is particularly intriguing, especially in light of the fact that HTRA1 has potential chaperone activity due to its C-terminal PDZ domains and has a preference for misfolded substrates (44). While more work needs to be done on the role this enzyme plays in tau proteolysis, these findings further indicate the complexity and likely involvement of multiple players in this process.

### Calpains

Calpains are calcium-activated cytosolic cysteine proteases. Two isoforms differentiated and named by their sensitivities to calcium (i.e., μ-calpain and m-calpain, also called calpain-1 and calpain-2) are abundant in the central nervous system, and respond to micromolar and millimolar concentrations of calcium, respectively (45). Calpain has been implicated in a number of neurodegenerative diseases [for a review, see (46)]. The active form of calpain-2 is found in 50–75% of NFTs in tauopathies including AD, but not in protein aggregates found in other diseases (47). This is consistent with another study that found equivalent calpain levels between control and AD cases, but the activity level of the enzyme isolated from AD brain tissue was increased (48). Excitotoxicity leading to elevated intracellular calcium is a common feature of neurodegenerative diseases, and is implicated in AD (49, 50). This process may lead to enhanced activation of calpains (51). This in turn could influence a number of pathologic processes, including tau proteolysis. Indeed, tau has a number of putative calpain cleavage sites, and incubation of recombinant tau with calpain generates specific fragments, including one that is ∼35 kDa and one that is ∼17 kDa (19, 20). Increasing intracellular calcium levels in PC12 cells leads to calpain-induced cleavage of tau (18). This may reflect a potential effect of excitotoxicity in AD. Inducing apoptosis in cerebellar granule cells yields calpain-mediated tau fragments, including a dominant ∼17 kDa fragment (17). Also, treating primary hippocampal neurons with pre-aggregated amyloid β (Aβ) led to the generation of tau fragments of ∼35, ∼24, and ∼17 kDa, which was blocked by addition of a calpain inhibitor (52, 53). Tau fragments of the same size were also found in AD brain tissue (19).

The pathological role of this calpain-cleaved tau is unclear. While some studies demonstrate toxicity resulting from calpain proteolysis of tau, other studies do not support this conclusion. On the one hand, expressing a 17-kDa fragment of tau based on calpain cleavage site mapping in hippocampal neurons led to neurite retraction and the appearance of varicosities after 48 h (52). Additionally, suppressing calpain activity in a fly model of tauopathy prevented neurodegeneration, as did expressing a calpain-resistant form of tau (54). In contrast, another study used mass spectroscopy and sequencing to identify the “17 kDa” tau cleavage product and found it did not correspond to the recombinant fragment utilized in the above studies (19). Expression of a recombinant form of the mass spectroscopy-identified fragment in hippocampal neurons was not toxic (19). Further studies are needed to clarify the contribution of calpain-mediated proteolysis of tau to AD pathology.

### Caspases

There is significant evidence that tau is a caspase substrate and that caspase-mediated tau cleavage may play a role in AD pathology. Early *in vitro* studies demonstrated that tau is cleaved in the C-terminus by several caspases including caspase-3 and caspase-6 (21–23). Caspase-6 was also shown to cleave the N-terminus of tau *in vitro* (24). Caspase-3, which is a key effector in the apoptotic cascade, cleaves tau predominantly at the C-terminal D421 site generating a fragment often referred to as tauC3 (22, 23). There may be reciprocity with the apoptosis pathway as activating caspase-3 by inducing apoptosis in cortical neuronal culture led to tau cleavage (22), and selectively expressing tauC3 led to apoptosis in NT2 and COS cells (21). This might represent a feed-forward loop of neurotoxicity. Furthermore, expressing a cleavage resistant form of tau (D421E) protects cells from apoptotic cell death (22). Another potential mechanism of inducing caspase-3 cleavage of tau is the presence of Aβ peptides. TauC3 is formed in primary cortical neurons after treatment with Aβ (23).

Caspase cleavage of tau may play a role in stimulating the tau aggregation seen in AD. Indeed, *in vitro* polymerization assays demonstrate that caspase-cleaved tau has a greater propensity to aggregate compared to full-length tau (23, 55). Intriguingly, caspase activation was shown to immediately and consistently precede the formation of tangles (56). This group used *in vivo* multiphoton imaging in Tg4510 TAU^P301L^ mice to simultaneously image activated caspases and Thioflavin-S positive tau tangles. There was a strong correlation between active caspases and the presence of tangles within viable neurons. In the few cells found that were caspase-positive and tangle-negative, 88% had tangles within 24 h (56). This seems to further support a role for caspase cleavage in the evolution of tau pathology.

In order for caspase to cleave tau in the AD brain, it needs to be present in its active form. The active forms of both caspase-3 and caspase-6 are elevated in AD-specific brain regions (temporal and frontal lobes) compared to unaffected regions (cerebellum) and control brains (57, 58). Furthermore, active caspase co-localizes to NFTs (58), and caspase-cleaved tau is found in AD-affected brain regions, particularly in neurons displaying tangle pathology (59, 60). This includes tau cleaved by caspase-6 in the C-terminus (58–60) as well as in the N-terminus (24). TauC3 is present in AD brain – in neurons and co-localized with NFTs – and inversely correlates with cognitive function (55, 60, 61).

The activation of caspases and the subsequent cleavage of tau is likely to occur independent of apoptotic cell death (56). The processes that may result in the activation of caspases in an apoptosis independent manner have not been clearly delineated; however several possibilities have been suggested. First, inflammation, which is a common feature of AD, may contribute to tau pathology by activating caspases. Treating cells with the prostaglandin cyclopentenone byproduct PGJ2 increased caspase activity and increased cleaved tau (62). Thrombin signaling can also activate caspases (36). Proteasomal impairment appears to be upstream of caspase activation, as inhibiting the proteasome with epoxomicin (EPX) led to activation of caspase-3 in primary neurons (63) and in a neuroblastoma cell line expressing wild-type tau (64). In both studies caspase activation correlated with the appearance and increase over time of caspase-cleaved tau species, which appeared to subsequently form aggregates in the neurons (63). While the mechanism is unclear, a possibility is that accumulating proteins might be a factor in initiating caspase activation.

## Proteolysis vs. Degradation

**Figure 1:**
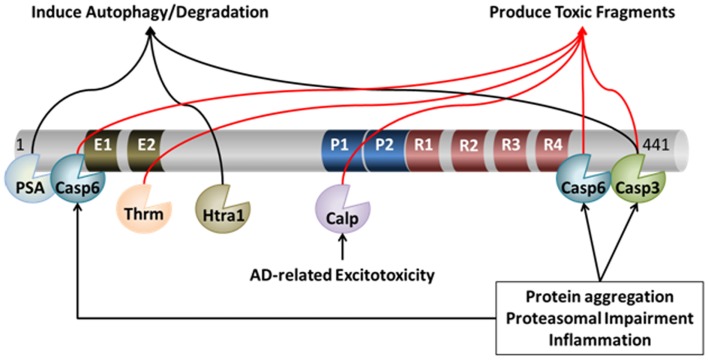
**Proteolytic processing of tau**. Under pathological and physiological conditions, tau undergoes cleavage at many distinct proteolytic sites by a myriad of proteases. The action of these proteases can lead to both protection and/or exacerbation of pathology. For example, cleavage of tau by caspase (Casp) 3, caspase-6, calpain (Calp), and thrombin (Thrm) leads to the production of toxic fragments of tau that exacerbate pathology. On the other hand, cleavage of tau by PSA, Htra1, and – in some circumstances – caspase-3, may facilitate its degradation, which may protect neurons from AD-related neuronal death.

As discussed above, a number of enzymes have been shown to act on tau, under potentially pathological, as well as physiological conditions. Many of these enzymes cleave tau at discrete sites, generating specific fragments. Some of these fragments, such as those generated by thrombin, calpain, and caspase, are potentially toxic to the cell if they accumulate due to inefficient clearance mechanisms. Figure 1 illustrates the potential contribution of these different proteases to the processing of tau. These proteolytically generated tau fragments can show an increased propensity for self-association, prior to the formation of overt aggregates. Thus, in the context of enhanced proteolysis (for example by caspases) there may be increased low-order oligomers formed by cleaved tau species. These oligomers may be unable to be cleared as effectively by the cell and contribute to neuronal dysfunction. Therefore coordination between proteolytic processing of tau and clearance by degradative pathways is essential for maintaining the appropriate levels of tau in a functional state. Below we will discuss the main degradative pathways of the cell-the proteasome and autophagy-which likely clear full-length tau as well as proteolytically generated tau fragments.

### The proteasome

**Figure 2:**
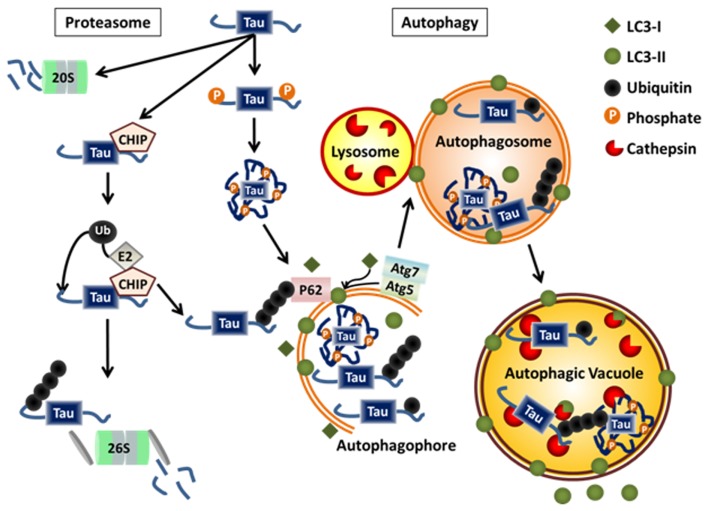
**Physiological degradation of tau**. Tau is degraded by both the proteasome and autophagy systems. Targeting of tau to either system may be determined by the extent and nature of post-translational modifications, the folding state, the level of aggregation, and its interaction with chaperone proteins or ubiquitin ligases. Monomeric tau is natively unfolded making it a likely target for the 20S proteasome. Monomeric tau also interacts with the E3 ligase, CHIP, which can lead to its ubiquitylation and degradation via the 26S proteasome or autophagy. Certain cleavage products and phosphorylated forms of tau, as well as, monoubiquitylated tau and tau aggregates are selectively degraded by autophagy.

The proteasome is a multimeric barrel-shaped structure that is a key complex for clearing soluble cytosolic proteins. The 26S proteasome has a regulatory cap (19S, or alternatively the 11S regulatory particle) on either end of its catalytic core (20S), which contains the proteolytic activities and degrades substrates tagged with poly-ubiquitin chains as the targeting sequence. The regulatory cap in conjunction with chaperone proteins unfolds the protein substrate and removes the ubiquitin tag in an ATP-dependent process prior to feeding the protein into the catalytic core, where it is systematically degraded by the enzymatic properties of the proteasome. The 20S proteasome, which is the catalytic core without its regulatory caps, is also able to degrade natively unfolded substrates directly through an ATP- and ubiquitin-independent process. As shown in Figure 2, tau is an ideal proteasomal substrate for either form of the proteasome because it is a relatively small, unfolded, short-lived cytosolic protein (64–67).

The accumulation of proteins in AD patients’ brains generated interest in the role of proteasomal function. There is evidence suggesting that proteasomal activity, but not protein level, is decreased in AD-sensitive brain regions specifically compared to unaffected regions (68, 69). Additionally, tau appears to be physically associated with the proteasome in brain tissue from AD cases. When tau was immunoprecipitated it pulled down both the 26S and 20S proteasomes, while immunoprecipitating for the 20S catalytic core pulled down tau (69). This suggests tau is being targeted to the proteasome, but may also indicate impaired ability to complete degradation; hence it is remaining associated with the proteasome. Further, there was an inverse correlation between proteasomal activity and high molecular weight forms of tau (69). This may suggest that abnormal proteins themselves may interfere with proteasomal degradative processes. Indeed, *in vitro* aggregated paired helical filament tau could inhibit proteasome activity (69).

### Evidence that tau is degraded by the proteasome

A number of studies have used various *in vitro* techniques to analyze proteasomal degradation of tau. These include cell culture and cell free studies. Not surprisingly, if recombinant tau is incubated with isolated 20S proteasomal complexes, degradation occurs (65). In this system proteolysis is bidirectional. Also, if tau is first ubiquitylated in an *in vitro* reaction and then incubated with isolated 26S proteasomes supplemented with MgCl_2_ and ATP, degradation proceeds (66). These data indicate tau can be a substrate for both forms of the proteasome. Similar data has been obtained from studies using various cell culture systems as well as animal tissue and primary cultures with a variety of proteasomal inhibitors. When HEK cells are co-transfected with tau and ubiquitin, tau accumulates in the insoluble fraction. Its accumulation in the insoluble fraction is enhanced by proteasomal inhibition (using ALLN or MG-132) suggesting that tau is degraded by the proteasome (66). In SH-SY5Y neuroblastoma cells, treatment with lactacystin, a selective inhibitor of the 20S catalytic core, maintained levels of transfected wild-type full-length tau (4R0N) after cycloheximide treatment halted protein synthesis (65). Similarly, overexpressing the FTDP-17 mutant P301L tau in SH-SY5Y cells and then treating with lactacystin led to significantly increased tau levels (70). Lactacystin also caused accumulation of endogenous tau in the HT22 murine neuronal cell line (71). In immortalized mouse cortical neuronal cells inducibly expressing full-length wild-type tau, EPX slowed the degradation of full-length tau (72). In M1C neuroblastoma cells that inducibly express wild-type full-length tau (4R0N), EPX, and MG-132 induced accumulation of full-length tau but there was a concomitant loss of C-terminus immunoreactivity (64). This was attributed to caspase cleavage, as activated caspase-3 was detected, and a caspase inhibitor preserved C-terminal immunoreactivity (64). Additionally, incubation of rat brain extract (containing endogenous tau and proteasomal enzymes) with the proteasome activators Mg^2+^ and ATP resulted in lower total tau levels with an increase in smaller forms, compared to extract not supplemented with Mg^2+^ and ATP (73). The loss of tau was blocked by lactacystin giving further evidence that the proteasome was degrading tau (73). The story is more complex, however, as proteasomal inhibition under physiological conditions does not consistently lead to tau accumulation. For example, treatment of primary neurons with an Hsp90 inhibitor to interrupt the proper chaperoning of tau leads to decreased levels of tau. Adding MG-132 to block the proteasome prevented the Hsp90 inhibitor-induced reduction in total tau. MG-132 alone had no effect on tau levels (67). This might suggest that under normal circumstances, if proteasomal impairment occurs, tau levels are maintained by autophagic degradation. But when the system is pushed to promote proteasomal degradation over autophagy – such as by inhibiting Hsp90 – then the homeostatic maintenance of tau levels is disrupted and tau degradation does not occur when the proteasome is inhibited.

### Autophagy

Autophagy is the process of “self-eating.” Under starvation conditions, bulk autophagy can be induced to catabolize cellular substrates to generate energy. However it is now evident that autophagy is an ongoing clearance mechanism for larger, longer-lived proteins and aggregates, as well as organelles such as mitochondria and peroxisomes (74) and pathogenic bacteria (75–77). There are three forms of autophagy: microautophagy, macroautophagy, and chaperone-mediated autophagy. The most common and well understood is macroautophagy, hereafter referred to simply as autophagy. For a more complete review of autophagy, see (78). Briefly, a double membrane autophagophore is initiated and subsequently expanded to engulf a region of cytoplasm containing the substrate/substrates to be degraded, such as tau (see Figure 2). Once fully formed into an enclosed vesicle called an autophagosome, it is trafficked to a lysosome where it undergoes fusion to become an autophagic vacuole (AV). The lysosomal enzymes degrade the inner membrane of the autophagosome as well as the delivered contents. The enzymes responsible for degrading protein substrates of autophagy are the cathepsins. Once the contents are fully degraded the lysosome is regenerated via acidification through vacuolar ATPases. There are 15 core autophagy related genes (Atgs) that are involved in the process of autophagy. Many of these have E1, E2, or E3 ligase activity to catalyze the reactions necessary for the initiation and expansion of the autophagosomal membrane. Critical early steps in the formation of the autophagopore require a complex of Atg proteins that conjugate phosphatidylethanolamine onto Atg8 family members (including LC3), a process that is critical for allowing expansion of the autophagosomal membrane. Conjugated LC3, called LC3-II, is the canonical marker of autophagosomes. Atg7 is a critical E1 ligase for several of the reactions necessary for autophagy (74).

### Evidence that autophagy is impaired in AD

There is significant support for the possibility of defective autophagy in AD. Electron microscopic analysis of brain tissue from confirmed AD cases revealed that AVs accumulated in dystrophic neurites and correlated with the presence of filamentous tau (79). However, this correlation was not quantified (79). Similar results were observed in mouse models of AD. For example, in a presenilin 1 (PS1)/Amyloid Precursor Protein (APP) double transgenic mouse, AVs were prevalent in dystrophic neurites at as early as 4.5 months without a similar accumulation of other structures such as lysosomes (80, 81). In these transgenic mice LC3-positive bodies were particularly apparent in neurites surrounding amyloid plaques, and immunoblotting of hippocampi from 6 month old transgenic PS1/APP mice revealed increased levels of LC3-II compared to wild-type mice (81). It is well established that mutations in PS1 result in familial AD, and until recently it was thought that this was only due to alterations in APP processing. However PS1 has a number of non-secretase functions, including acting as the chaperone for the vacuolar-ATPase used to acidify the lysosomal lumen (82, 83). Mutations in PS1 were shown to impair the acidification of lysosomes, which is necessary for activating the proteolytic enzymes in this compartment. Improper acidification and impaired proteolysis of substrates would compromise the autophagy system and result in the accumulation of AVs as described above. However, another mouse model, the TgCRND8 mouse, which expresses mutant APP only, also has increased staining for LC3-II, as well as an increase in cathepsin D-positive lysosomes (84). This demonstrates that in the absence of mutant PS1, AD-associated impairment in autophagy occurs and thus is due to other factors. Treatment of *ex vivo* hippocampal slice cultures with lysosomal disruptors causes the formation of enlarged, dystrophic neurites filled with AVs and lysosomes, similar to what is seen in mouse AD models and human AD tissue (85, 86). It has also been suggested that specific cathepsins may become extralysosomal in certain diseases, including AD (87, 88). Together these observations implicate a possible failure of autophagy as part of AD pathogenesis.

### Evidence tau can be degraded by autophagy

As indicated above, a functioning lysosomal compartment is critical for the completion of autophagy. Given the possibility of a defect at this level of autophagy, numerous studies have directly assessed the effects of impairing lysosomal function on tau turnover, including specifically targeting the cathepsins. In an early study the direct cleavage of tau by cathepsin D was investigated in an *in vitro* assay using tau partially purified from rat brain in combination with cathepsin D from human liver. Incubation of tau with cathepsin D at pH 4.0 resulted in a decrease in full-length tau and a concomitant increase in cleaved fragments of varying sizes (89). Similarly, adding exogenous cathepsin D to homogenates of rat cortex at a neutral pH also generated tau fragments. Intriguingly, if a cysteine protease inhibitor was added to the assay, tau cleavage stopped at the 29-kDa fragment, suggesting that cathepsin D (an aspartyl protease) could cleave tau to a 29-kDa fragment after which other proteases may act to further degrade the protein. This also suggests if cathepsin D was able to escape from the lysosome, for example in the context of an AD-related stressor, it could still function in the neutral environment of the cytosol. However, the activity of cathepsin D at the more neutral pH may be more impeded than appears, as a previous study found cathepsin D’s proteolytic activity was significantly reduced above pH 6.0 (90). Treating hippocampal slices with chloroquine (CQ), which raises the pH of lysosomes to impair enzymatic function, was associated with increased levels of full-length tau (89, 91). This was in conjunction with an accumulation of intracellular PHF1 immunopositive tau (91). In M1C neuroblastoma cells that inducibly express full-length wild-type tau (4R0N), treatment with CQ also significantly slowed down tau degradation, and caused its accumulation (92). Treatment of hippocampal slices with the cathepsin modulator ZPAD (which stimulates cathepsin D very strongly) appears to increase the proteolysis of full-length tau resulting in the production of smaller fragments, including a phosphorylated 29 kDa fragment (86, 89). This partial degradation of tau was inhibited by inclusion of a selective cathepsin D inhibitor (86). Cathepsin D seems particularly important for degrading tau, as its expression was neuroprotective in a *Drosophila* tauopathy model. Levels of cathepsin D are elevated in flies expressing mutant human tau. If cathepsin D is genetically ablated, these tau flies exhibit enhanced neurotoxicity and a shorter lifespan (93).

Modulating autophagy through other approaches also indicates that tau can be degraded through this pathway. Overexpressing only the repeat domain of tau containing an FTDP-17 mutation in neuroblastoma cells leads to tau aggregation as well as the appearance of smaller proteolytic fragments. Using the autophagy inhibitor 3-methyladenine (3-MA) to block the formation of autophagosomes led to an increase in both soluble and insoluble tau (94). Directly activating autophagy through a variety of mechanisms leads consistently to enhanced tau clearance – either pathological forms or total tau. In a hippocampal slice preparation methylene blue was used to induce autophagy, which resulted in a decrease in phosphorylated tau and insoluble tau, specifically (95). In a cell line expressing the repeat domain of tau containing the FTDP-17ΔK280 mutant, treatment with the disaccharide trehalose, an mTor-independent autophagy activator, significantly reduced aggregated tau as measured by Thioflavin-S staining, as well as total tau levels both soluble and insoluble as detected by western blotting (96). Stimulating autophagy either through serum withdrawal or rapamycin treatment in SH-SY5Y cells overexpressing P301L tau that had been induced to aggregate led to substantial reduction in aggregates that was prevented by 3-MA (70). In a mouse model expressing the FTDP-17 mutant P301S, promoting autophagy with trehalose treatment beginning at weaning significantly reduced insoluble tau, as well as tau phosphorylated at T212/S214 (AT100) (97). However, no other phosphorylation sites were assessed. This effect was correlated with improved neuronal survival in cortical layers I–III (97). Stimulating autophagy via genetic manipulation of the mTor pathway decreased total and phosphorylated tau in the same mouse model (98). Conversely, inhibiting autophagy (also via mTor) lead to increased total and AT8-positive phosphorylated tau (98). Mice in which the critical autophagy gene Atg7 is knocked out in forebrain neurons develop age-dependent neurodegeneration with accumulation of phosphorylated tau within intracellular inclusions (99). These inclusions specifically contained tau phosphorylated at AT8, AT100, and TG3 epitopes, but not PHF1. Significantly, if tau was also knocked out in these autophagy-deficient mice, neurodegeneration was reduced (99).

Interestingly, other evidence for the role of autophagy in clearing tau was the result of attempting to elucidate the role of the proteasome in tau degradation. Treating rat primary neurons with the proteasomal inhibitor MG-132 actually led to a reduction in total tau. This effect was likely due to a compensatory upregulation of autophagy, as evidenced by increased LC3-II protein and an increased number of autophagosomes in treated cells (96). This will be discussed in more detail below, as it has important ramifications for the intersection of these two degradative pathways.

## Interplay between Autophagy and the Proteasome

There is compelling evidence for significant and extensive interplay between the autophagy and proteasomal systems. This has intriguing implications for disease processes and specifically tau degradation in AD. First, while each system preferentially degrades specific substrates, there are many substrates that can be degraded by both systems, tau being a prime example (25). For instance, a particular substrate may be degraded by the proteasome under normal conditions, but if that system is impaired and/or there is an excess of that substrate it may be degraded in a compensatory manner by autophagy. Another possibility is that particular forms of a substrate may be shuttled to one pathway or another. In the case of tau, as a monomer it is natively unfolded and hence a likely proteasomal substrate, as discussed above. However, any of the numerous modifications tau undergoes during AD pathogenesis may render it less able to do so, for example, by inducing conformational changes to a more ordered structure as suggested by several conformation-specific antibodies that label tau in AD brain (Alz-50, MC-1, etc.). Additionally, oligomerized or aggregated tau may not be a preferred proteasomal target. It has been suggested that this change in state is part of the signal for tau to be degraded by autophagy. This is supported by evidence that full-length tau, which has a lower propensity for aggregating, is cleared by the proteasome while caspase-cleaved tau, which is more aggregate prone, goes through autophagy (72). Also, aggregated tau can be cleared by inducing autophagy (70, 96).

Ubiquitin is implicated in targeting substrates to both pathways. Historically, poly-ubiquitin chains generated through lysine 48 (K48) linkages were viewed as the prototypical proteasomal targeting sequence, while K63 chains appeared more specific for autophagy. However, the experimental evidence indicates a more complex picture. For example, if HEK cells are transfected with tau and ubiquitin, tau is readily ubiquitylated and degraded by the proteasome (66). However, if a ubiquitin construct containing a site mutation at K63 is transfected in, no ubiquitylation of tau occurs. Other mutated forms of ubiquitin, including ubiquitin unable to form linkages at K48, still resulted in tau ubiquitylation. This indicates that in this experimental overexpression paradigm, K63 linkages are the primary ubiquitin linkage for tau, and they target tau to the proteasome rather than to autophagy (66). However, another study, also using HEK cells and ubiquitin K48 and K63 mutants, demonstrated that in the presence of the E3 ligase CHIP, tau could be ubiquitylated by both K48 and K63 linkages (100). The likelihood that *in vivo* tau can be ubiquitylated in multiple ways is supported by studies showing tau isolated from NFTs in human brain has several forms of ubiquitin linkages as well as mono-ubiquitylation (101, 102). These data suggest that the physical structure of the ubiquitin chain is unlikely to be a sufficient signal for selectively targeting tau to either the proteasome or autophagy. An alternate mechanism for specifically targeting substrates is the involvement of chaperone proteins. The chaperones involved in proteasomal targeting are not well characterized, although it is known that ubiquitin-tagged substrates are trafficked to the organelle. Currently identified chaperones include p62 and Hsp70 (66, 100). Slightly more is known about autophagy adaptors, and there is significant overlap, as both p62 and Hsp70 are adaptors for this pathway as well (103, 104). This further complicates the understanding of how a substrate is selectively targeted to one path or the other. For example, a ubiquitylated substrate can be bound by p62 and either delivered to the proteasome (66) or engulfed by an autophagosome via p62 binding to LC3 (105). These findings suggest the involvement of a currently unidentified chaperones and/or targeting signals, or undetermined additional factors.

Other characteristics of the substrate are likely to also play a role in successfully targeting the protein either to the proteasome or to autophagy. In the case of tau, two modifications seem to be critical for this process: phosphorylation and truncation. For example, in the study where rat brain extract was incubated with Mg^2+^ and ATP, there was an overall decrease in tau due to proteasomal activity; however tau phosphorylated at the PHF1 and Tau-1 epitopes seemed to be preferentially degraded as they were non-detectable within 3 h (73). The preferential degradation of specific phospho-forms of tau by a particular pathway has been reported in other studies as well. In CHO cells overexpressing P301L mutant tau, treatment with the Hsp90 inhibitor geldanamycin led to a more pronounced proteasome-mediated reduction in tau phosphorylated at proline-directed S/T sites compared to total tau (67). However, the levels of tau phosphorylated at KXGS sites within the repeat domain were not reduced by geldanamycin treatment. In agreement with those findings, inhibiting autophagy in primary rat cortical neurons with 3-MA resulted in the selective accumulation of tau phosphorylated at the KXGS motif S262 (recognized by the 12E8 antibody) (106). Additionally, in a hippocampal slice preparation, induction of autophagy by treatment with methylene blue led to a decrease in phosphorylated tau and insoluble tau without an effect on total tau (95). Activating autophagy with trehalose in rat cortical neurons demonstrated certain phospho-epitopes (AT8, PHF1, and 12E8) were reduced more significantly than total tau – up to 80% compared to the 20% reduction in total tau (96). Finally, caspase-3 cleaved tau has a shorter half-life than full-length tau and is preferentially degraded by autophagy (72). Additional modified forms of tau have yet to be fully examined for their preferred route of degradation.

As specific substrates are targeted to one degradative pathway or the other, the function of each system can also directly impact the functioning of the other. It is well documented that blocking the proteasome with small molecule inhibitors causes an increase in autophagic flux (107). This can be seen both as increased autophagosome formation and maturation as well as enhanced degradation of autophagy substrates (96). However the converse is not true; autophagy impairment does not elevate proteasomal function and, in fact, rather strikingly inhibits it. There are several possible mechanisms for this inhibition. The accumulation of large aggregated substrates might impair the proteasome, as seen for PHF tau (69). Also, reduced recycling of p62 by impairing autophagy (causing its accumulation) will impair proteasomal processing, potentially by p62 competing with other chaperones for proteasomal targets and impeding their delivery (107). The degradation of tau is thus a complex process mediated by multiple factors. While much is known about how tau can be cleared, additional studies are needed to clarify what actually happens in both the normal brain and in the context of AD. This information will yield critical insights into potential therapeutics.

## Role of Oligomers in Affecting Tau Degradation Decisions

**Figure 3:**
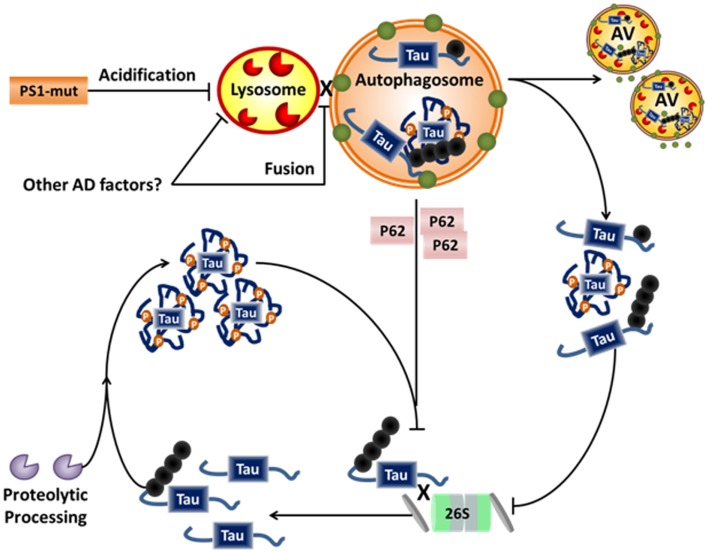
**Alzheimer disease-related disruption of tau degradation**. Impairment of protein degradation is a known component of both familial and sporadic AD. Familial AD-related mutations of PS1 are linked to impairment of lysosomal acidification and/or fusion to the autophagosome, while sporadic factors leading to similar impairments have not yet been elucidated. Impairment of the lysosome/autophagosome leads to accumulation of dysfunctional autophagic vacuoles (AVs), cytosolic p62, and aggregates of tau. Dysfunction of autophagy further inhibits the proteasome, possibly via accumulation of p62, which can be a chaperone for both systems. P62 is usually recycled via autophagy and accumulates in the cytosol when autophagy is impaired. Hypothetically, this allows p62 to compete with other proteasome chaperones thus inhibiting proteasomal degradation. Further, accumulation of aggregated proteins has also been shown to inhibit the proteasome. Taken together, this could lead to accumulation of soluble tau, and thus more proteolytic processing and further aggregate. Hence, a vicious cycle of degradative pathway impairment and tau accumulation/aggregation may contribute to the neurodegenerative processes in AD.

Given the data indicating tau can be processed by both autophagy and the proteasome, and furthermore that the signaling mechanisms directing substrates to either path are shared, it is unclear how decisions regarding which way tau is degraded are made. One possibility could be tau’s physical state of oligomerization. Soluble, monomeric tau is an ideal proteasomal substrate. Indeed, it has been clearly demonstrated that tau can be degraded by the proteasome (65–67, 73). It thus can be suggested that under physiologic circumstances much of tau is degraded in this manner, with select modified forms being cleared by autophagy. However, within the context of the AD milieu, additional tau modifications and degradative impairments may cause the balance to shift away from proteasomal degradation toward autophagy. For example, as discussed above, certain modified forms of tau, such as caspase-cleaved tau, have a stronger tendency to aggregate. As tau begins to assemble into oligomers, it may become increasingly undesirable as a proteasomal substrate. These low-order, soluble oligomers may be preferentially degraded by autophagy. However, as previously discussed, autophagy is likely impaired in AD. The tendency for certain phospho-epitopes to show preferential clearance by certain pathways may also relate to their propensity for aggregation. As tau oligomers increase in size, density, and modifications during the development of filaments and tangles, not only will they be unable to undergo proteasomal degradation, they may directly impair proteasomal function (69). This proteasomal impairment could have multiple effects. For example, autophagy may initially be activated as a compensatory response. Caspase-3 and possibly other proteases may also be activated as well. However, this may result in an accumulation of potentially toxic cleaved forms of tau. Additionally, given the significant evidence that autophagy is impaired in AD, possibly at the level of the lysosomes, proteasomally mediated activation may serve to further obstruct the autophagy system (see Figure 3).

## Conclusion

It is clear that tau plays a significant role in AD pathology, although the mechanisms involved have not been clearly delineated. Tau is a normal neuronal protein that modulates microtubule-based functions, and becomes increasingly hyperphosphorylated, truncated, and otherwise modified in AD. These modifications not only impair tau’s normal function, but also appear to promote its oligomerization. These oligomers eventually accumulate to form the NFTs which are pathognomonic for AD. While the NFTs may be harmful to the cell in some ways, it is now believed that the principal toxicity results from pre-aggregated, soluble tau oligomers. Thus, understanding how these tau species can be cleared may allow for the development of effective therapeutic approaches. It is clear from the data that certain species of tau are preferentially degraded by the proteasome and others by autophagy. There is evidence that both of these degradative systems are likely impaired at some level in AD. Additionally, there is a complex interplay between the proteolytic and degradative pathways that suggests a cycle of pathology may develop in AD whereby alterations in tau processing, including by cytosolic proteases, pushes more tau toward the autophagy system. Decreased autophagic function would result in accumulation of these autophagy-cleared tau species. The combination of impaired autophagy and accumulating substrates has the potential to lead to proteasomal inhibition, in addition to other factors (such as Aβ) that may impair the proteasome in AD (108). This then further promotes cytosolic accumulation of tau leading to its aggregation. Additionally, modifications including caspase-3 cleavage and hyperphosphorylation promote aggregation even of full-length tau, reducing the pool of functional tau.

Understanding how tau is cleared may enable us to identify potential mechanisms for enhancing clearance of pathological forms of tau. Ameliorating the deficit in autophagy is a likely target for this process, and initial results of stimulating autophagy show promise for clearing tau. Indeed, several studies aimed at stimulating autophagy have demonstrated efficacy in reducing phosphorylated and aggregated tau in both *in vitro* and *in vivo* models (95–98). These studies are an important initial step toward elucidating the exact role of tau degradation in modulating neurodegeneration in AD. Further studies that better gauge the contribution of each degradative pathway will be necessary. Due to the complexity of the cellular environment, *in vitro* studies that can tightly control for variables including tau modifications and proteolytic pathway function will likely be instrumental. Ultimately, a more complete understanding of the differential contribution of various proteolytic and degradative pathways will provide critical opportunities for therapeutically addressing the tau pathology associated with neurodegeneration in AD.

## Conflict of Interest Statement

The authors declare that the research was conducted in the absence of any commercial or financial relationships that could be construed as a potential conflict of interest.
